# The initial intravenous treatment of a human immunodeficiency virus-infected child with complicated abdominal tuberculosis

**DOI:** 10.4102/sajhivmed.v21i1.1121

**Published:** 2020-08-24

**Authors:** Anthony K. Enimil, Brian Eley, James Nuttall

**Affiliations:** 1 Department of Paediatrics and Child Health, College of Health Sciences, University of Cape Town, Cape Town, South Africa; 2 Department of Paediatrics and Child Health, College of Health Sciences, Red Cross War Memorial Children’s Hospital, Cape Town, South Africa

**Keywords:** intravenous, antituberculosis, tuberculosis, child none, ARV medications

## Abstract

**Introduction:**

There is very limited published experience with intravenous (IV) antituberculosis (anti-TB) and antiretroviral therapy (ART) especially in children. We have described a human immunodeficiency virus (HIV)-infected child with complicated abdominal tuberculosis who was initially treated with IV anti-TB and a partially IV ART regimen before transitioning to oral therapy.

**Patient presentation:**

A 3-year-old boy presented with hypovolaemic shock with a 3-day history of inability to pass stools, abdominal distension and bile-stained vomiting. Abdominal ultrasound and X-ray showed small-bowel obstruction. Human immunodeficiency virus antibody testing was positive, and Cluster of Differentiation (CD)4+ lymphocyte count was 56 cells/mL (15%). Xpert *Mycobacterium tuberculosis* (MTB)/Rifampicin (RIF) Ultra and TB culture on induced sputum detected MTB complex sensitive to rifampicin and isoniazid.

**Management and outcome:**

Following laparotomy and closure of bowel perforations, the child was commenced on IV rifampicin, moxifloxacin and amikacin. Amikacin was stopped after 3 days because of nephrotoxicity, and meropenem and IV linezolid were added. After 20 days, ART comprising IV zidovudine, oral lamivudine solution, oral lopinavir/ritonavir solution and additional oral ritonavir solution for super boosting was commenced. By day 40, the patient was well established on oral feeds and was switched to standard oral anti-TB medications. Sputum examined 1 month after starting the treatment was found culture-negative for MTB. After 4 months of treatment, the HIV viral load was < 100 copies/mL. He completed a total of 12 months of anti-TB treatment.

**Conclusion:**

Despite limited experience and few available IV formulations of standard anti-TB and ARV medications, initial IV therapy may be beneficial for patients in whom oral medication is not an option.

## Introduction

There is limited published experience with the use of intravenous (IV) antituberculosis (anti-TB) drugs in patients with severe tuberculosis (TB). We describe the challenges of managing a human immunodeficiency virus (HIV) and TB co-infected child with complicated abdominal TB in whom oral treatment was not initially feasible. The aim of this report was to highlight the role of IV therapy in this context.

## Patient presentation

A 3-year-old boy presented with a 3-day history of inability to pass stools, abdominal swelling and bile-stained vomiting. His mother had a history of substance abuse. The pregnancy and delivery had been un-booked. She was diagnosed HIV-positive post-partum and started on antiretroviral therapy (ART). Infant antiretroviral prophylaxis was prescribed for the child. It is not known whether HIV testing of the infant at the time of birth was performed. The mother defaulted her ART and her infant’s ARV prophylaxis. The child had no history of contact with a TB source case.

**FIGURE 1:**
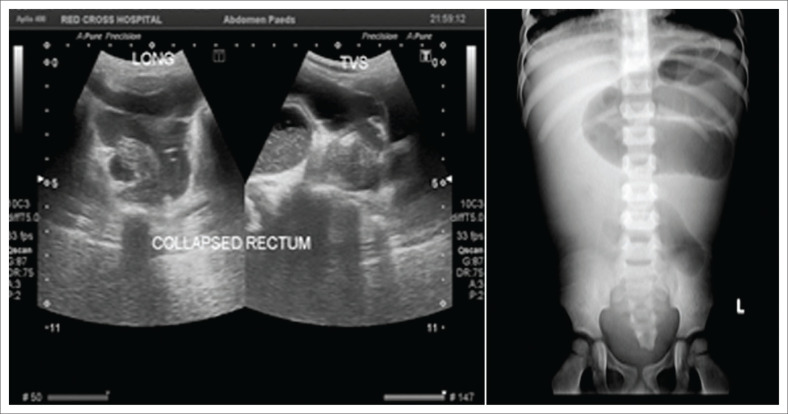
Ultrasound and supine abdominal X-ray showing markedly distended small bowel loops and collapsed rectum.

On examination, the child had abdomen distension. He was in shock, pale and severely underweight. The HIV antibody test was positive on two separate samples. The CD4+ lymphocyte count on admission was 56 cells/mL (15%), haemoglobin (Hb) was 5 g/dL and the platelet count was 114 × 10^9^/L. The chest X-ray did not suggest pulmonary TB. Sputum gene Xpert *Mycobacterium tuberculosis* (MTB)/RIF Ultra was positive for rifampicin-sensitive MTB. The sputum culture grew rifampicin- and isoniazid-sensitive TB. Abdominal radiography and ultrasonography showed distended small-bowel loops with no gas in the rectum, consistent with small-bowel obstruction (Figure 1).

## Management and outcome

The child was given a blood transfusion and was resuscitated. At laparotomy 3 days after admission, three small-bowel perforations with faecal soiling of the abdominal cavity and ‘caseous areas’ of the ileum, ascending colon, mesenteric and retroperitoneal lymph nodes were identified. The abdomen was washed with saline and the perforations closed. Histology of the intra-abdominal nodes showed necrotising granulomatous inflammation.

The child was commenced on IV rifampicin, IV moxifloxacin and IV amikacin. On day 3, his creatinine concentration increased from 61 µmol/L to 133 µmol/L. Amikacin was stopped and moxifloxacin dose was adjusted according to the estimated glomerular filtration rate (eGFR) of 26.6 mL/min/1.73 m^2^. Intravenous meropenem and IV linezolid (LZD) were started. The patient’s renal function normalised in the following 12 days. The platelet count and the Hb levels declined, and LZD was discontinued after 10 days. On day 20, ART, namely, IV zidovudine (ZDV or AZT), oral lamivudine (3TC) solution, oral lopinavir or ritonavir (LPV/r) solution and oral ritonavir solution to ‘super-boost’ the LPV, was introduced. The plasma LPV ‘trough’ concentration 1 week after starting the ART was therapeutic, namely, C_min_ 9.98 mg/L. By day 30, he was tolerating small oral feeds. Intravenous meropenem was stopped and oral isoniazid was started. On day 40, he was settled on oral feeds, and oral rifampicin/isoniazid, pyrazinamide and ethambutol tablets replaced the IV rifampicin and IV moxifloxacin. Intravenous AZT was replaced with abacavir. Liver transaminases had not been significantly altered by the TB therapy or the ART. Four weeks after starting TB treatment, sputum culture was found negative for MTB. Four months after starting ART, the HIV viral load was < 100 copies/mL, and at 12 months the CD4 count was 1111 cells/mL (25%). In total, the patient received approximately 11 months of oral anti-TB therapy. His weight-for-age *Z*-score 3 months after the completion of the TB treatment was within the normal range, that is, above –2. This had been less than –3 before starting the treatment.

## Discussion

This severely immunocompromised HIV/TB co-infected young child diagnosed with pulmonary and abdominal TB complicated by intestinal obstruction and perforation was treated with a modified, initially IV anti-TB treatment regimen before transitioning to standard oral drug-sensitive anti-TB treatment and ART. Notwithstanding the drug-associated adverse events, this initial strategy allowed the immediate provision of TB therapy while awaiting the return of normal gastrointestinal function, and this has been likely to have contributed to the positive clinical outcome.

The literature on the role of IV anti-TB treatment in patients who are unable to take oral medication is, as far as we are aware, limited to a few adult case reports.^1,2^ Intravenous formulations of anti-TB medications currently available in South Africa include rifampicin – this requires approval from the South African Health Products Regulatory Authority (SAPHRA) – moxifloxacin, levofloxacin, LZD, meropenem, imipenem/cilastatin and amikacin. Oral fluoroquinolones, oral LZD and IV carbapenems in combination with IV amoxicillin–clavulanic acid are also used in some rifampicin-resistant TB treatment regimens. Amikacin is no longer recommended except in extremely drug-resistant cases with few alternatives.^3,4^ Our patient was treated with amikacin prior to the release of these recommendations. Intravenous meropenem was used without recourse to amoxicillin–clavulanic acid. Levofloxacin was unavailable but because of less effect on Corrected QT interval (QTc) interval prolongation, it is preferred to moxifloxacin.^5^ Nephrotoxicity because of amikacin and haematological toxicity because of LZD resulted in the discontinuation of these drugs in our patient.^6,7^ The duration of IV anti-TB therapy was 40 days in this patient. Other case reports have described 40–60 days’ duration of injectable anti-TB treatment with or without concomitant oral anti-TB medication.^1,2^

## Conclusion

Intravenous anti-TB treatment must be considered in patients with complicated abdominal TB in whom oral intake is not feasible. The optimal combination of medication is uncertain and close monitoring of treatment efficacy and toxicity is essential.
